# Evaluation of the early effects of peptide-containing agents in sensitivity treatment regarding their structural, morphological, and elemental changes in dentin tissue and their penetration efficacy

**DOI:** 10.1590/1678-7765-2026-0127

**Published:** 2026-06-12

**Authors:** Tugba Ozdemir, Selcuk Savas, Ebru Kucukyilmaz Izgi

**Affiliations:** 1 Izmir Katip Celebi University Faculty of Dentistry Department of Pediatric Dentistry Izmir Turkey Izmir Katip Celebi University, Faculty of Dentistry, Department of Pediatric Dentistry, Izmir, Turkey

**Keywords:** Dentin hypersensitivity, Biofunctional materials, Self-assembling peptides, SPRG

## Abstract

**Correspondence:**

Ebru Kucukyılmaz Izgı - Izmir Katip Celebi University - Faculty of Dentistry - Department of Pediatric Dentistry – Cigli – Izmir – Turkey - E-mail: ebrukucukyilmaz@hotmail.com - Tel: +902323524040

**Purpose:**

This study aimed to evaluate the effects of various therapeutic agents on dentin tissue in the management of dentin hypersensitivity.

**Methodology:**

Overall, 30 caries-free human third molars were sectioned into dentin specimens (n=120) and randomly assigned to control or treatment groups (Curodont™ D’Senz, PRG Barrier Coat, and Curodont™ Repair). After 24 h of artificial saliva storage, dentin microhardness, surface morphology, open dentin tubule count, elemental composition, and penetration depth were evaluated using Vickers microhardness testing, SEM/EDS, and CLSM. Statistical analyses were conducted with a 0.05 significance level.

**Results:**

The microhardness measurements showed a statistically significant increase in all groups in comparison with the control group (p<0.05), with no significant differences between the treated groups (p>0.05). EDS analysis showed statistically significant differences between the groups, with the highest calcium and phosphorus levels observed in the PRG Barrier Coat group (p<0.05). SEM analysis showed that the PRG Barrier Coat treatment resulted in the most extensive tubule occlusion and the smallest open tubule diameters (p<0.05). Curodont™ D'Senz and PRG Barrier Coat showed the most significant tubule penetration depths and the highest laser fluorescence values, as per CLSM analyses (p<0.001).

**Conclusion:**

The findings suggested that the tested materials may occlude dentinal tubules and penetrate dentin to varying extents, with PRG Barrier Coat showing the most pronounced effect. Self-assembling peptide-based agents show limited short-term efficacy but offer potential for biomimetic remineralization.

## Graphical abstract

**Figure:**
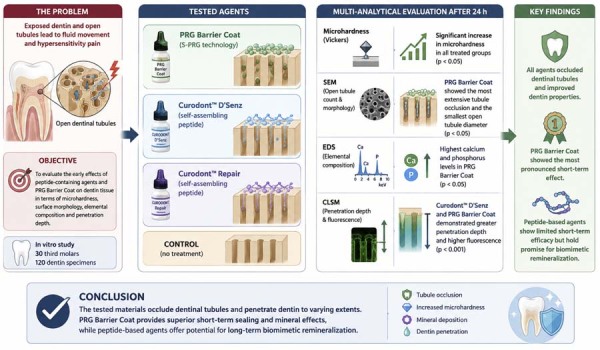

## Introduction

Dentin sensitivity is a common condition characterized by short-term, sharp pain in response to chemical, thermal, tactile, or osmotic stimuli. It affects an average of 33% of people worldwide, negatively impacting their quality of life.^1^ It is commonly observed in adults, particularly in conjunction with periodontal disease, whereas, in children, it is associated with certain developmental dental anomalies, early childhood caries, and dental erosion.^2^

The most widely accepted mechanism for the development of dentin sensitivity-related pain is the hydrodynamic theory. According to it, fluid movement within the dentin tubules caused by external stimuli stimulates the nerve endings at the pulp/dentin border, resulting in pain.^3^The literature reports that tubules are generally occluded in healthy dentin surfaces or those without sensitivity symptoms; in contrast, tubules are wider and more open in individuals with sensitivity symptoms.^4^ Therefore, a strong relationship has been showed between tubule occlusion and the alleviation of dentin sensitivity symptoms.^4^ For this reason, the physical closure of tubule openings or the promotion of mineralization by agents applied to the dentin surface is considered a fundamental mechanism of action to control dentin sensitivity.

A variety of agents (including corticosteroids, glutaraldehyde, fluoride, potassium oxalate, CPP-ACP, and arginine-calcium carbonate complexes) have been developed to reduce dentin sensitivity.^5,6^ These agents mainly aim to provide micromechanical occlusion by creating a physical barrier in the dentinal tubules or promoting mineralization, thereby targeting the hydrodynamic theory. However, most of these materials neither deliver rapid and adequate results due to their limited surface effects nor achieve deep dentin penetration, lacking resistance to mechanical stress over the long term.^5,6^

The recent development of PRG Barrier Coat represents a significant advancement in the field of biologically active giomer-based resin materials, with its formulation being achieved via pre-reacted surface-active glass ionomer (S-PRG) technology.^7,8^ The manufacturer asserts that the material is deemed suitable for areas showing a high risk of caries and widespread dentine sensitivity. This assertion is supported by the high ion release, fluoride recharge capacity, buffering ability in acidic environments, and mechanical stability of the material.^8,9^ In the treatment of dentin sensitivity, biomimetic remineralization-based approaches are also being developed as alternatives to classical desensitizing agents. In this context, agents containing self-assembling peptide matrices hold particular interest.^10-13^ Curodont™ D'Senz and Curodont™ Repair are biomimetic products based on oligopeptide 103 and P11-4-based Curolox^®^ technology, containing self-assembling peptides. Although Curodont™ D’Senz and Curodont™ Repair are often grouped under the same category as self-assembling peptide–based biomimetic agents, they differ substantially in formulation, intended clinical indication, and proposed mechanisms of action. Curodont™ Repair is primarily designed to promote biomimetic remineralization by forming a peptide-guided scaffold that facilitates calcium and phosphate deposition, whereas Curodont™ D’Senz is intended for dentin hypersensitivity management, and is suggested to act via rapid interaction with exposed dentinal tubules, forming a protective peptide-based layer that limits fluid movement.^10-14^ However, despite sharing self-assembling peptide technology, the dentin-level effects of these materials are unable to be taken to be identical as peptide behavior highly depends on the target tissue, local environment, and application conditions. The current literature predominantly evaluates peptide-based systems or S-PRG–based coatings using isolated outcome measures, such as tubule occlusion or mineral changes, often under heterogeneous experimental protocols. Consequently, it remains unclear whether their clinical benefits arise from surface sealing, true mineral reinforcement, intratubular penetration, or a combination of these mechanisms. Moreover, reducing peptide-based technologies to a single mode of action—such as remineralization or tubule occlusion alone—may underestimate their complex and dynamic interactions within a structurally heterogeneous tissue such as dentin.

Dentin hypersensitivity causing immediate and sharp discomfort upon exposure to stimuli has increased the clinical demand for treatment options that can rapidly alleviate symptoms. Despite the availability of multiple desensitizing approaches, standardized *in vitro* evidence evaluating the concurrent changes in (i) surface microhardness (as a proxy for mechanical reinforcement), (ii) dentinal tubule occlusion morphology, (iii) elemental/mineral profile, and (iv) intratubular penetration behavior remains limited. Therefore, this study aimed to investigate the short-term (24 h) effects of PRG Barrier Coat, Curodont™ D’Senz, and Curodont™ Repair on human dentin using Vickers microhardness, scanning electron microscopy (SEM) with energy-dispersive X-ray spectroscopy (EDS), and confocal laser scanning microscopy (CLSM) after 24-h artificial saliva storage. The null hypothesis in this study was defined as follows: There is no statistically significant difference in the effects of different agents used in sensitivity treatment on dentin tissue.

## Methodology

This study was conducted in accordance with the ethical guidelines of the Declaration of Helsinki and was approved by the Health Research Ethics Committee of Izmir Katip Celebi University (report number 2024-0243). Prior to it, a size analysis was performed on G*Power (version 3.1.9.2, Germany) based on a previous study.^15^ The required sample size was calculated with 80% power, a significance level of α = 0.05, and an effect size of 0.23, which indicated a total of 30 teeth specimens (120 dentin slices).

### Materials

**Figure 1:**
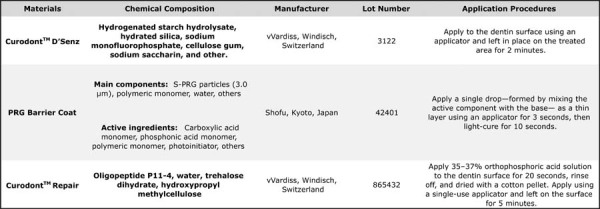
Chemical composition, manufacturers, and lot numbers and application procedures of the tested materials

In this study, three materials were used: PRG Barrier Coat, Curodont^TM^D’Senz, and Curodont^TM^Repair. The chemical composition, manufacturer, and batch numbers of the tested materials are shown in **Figure 1**.

* S-PRG: surface pre-reacted glass

### Specimen preparation

In this study, 30 extracted human third molars with no clinically visible abnormalities were subjected to a thorough cleaning using fluoride-free pumice to remove any extraneous debris or stains. Then, the teeth were stored in a 1% thymol solution, maintained at a temperature of 4°C, and used within one month of extraction. The teeth were rinsed and examined under a stereomicroscope (Olympus SZ61, Olympus Optical Co., Tokyo, Japan). Teeth showing defects, erosions, or microcracks were excluded from this study, whereas teeth with laser fluorescence scores of 0-5 (as measured by a DIAGNOdent™ pen, manufactured by KaVo, USA) were included. The roots of each tooth (n=30) were removed by sectioning approximately 1 mm below the cemento-enamel junction perpendicular to the long axis using a water-cooled diamond disc (Buehler Inc., Lake Bluff, IL, USA). In the subsequent stage of the procedure, the occlusal-coronal one-third of the crown portion was removed to expose the dentin surface. The crown structures obtained were then sectioned into four equal segments in the mesiodistal and buccolingual directions from the occlusal to the cervical regions so that the specimens allocated to the three experimental groups and the control group originated from the same tooth. This split-tooth design ensured that specimens allocated to the three experimental groups and the control group originated from the same tooth, minimizing biological variability among groups. Each dentin specimen (30 teeth, 120 dentin specimens) was embedded in self-curing acrylic resin (Integra, Ankara, Turkey) with the occlusal surface facing upward. The dentin surfaces of each tooth were then polished on a polishing machine (PRESI Mecatech 234, Tavernolles City, France) using 600-, 800-, and 1200-grit aluminum oxide abrasive paper to obtain a flat and smooth dentin surface. The prepared specimens were examined under a stereomicroscope to assess surface smoothness and identify the presence of any potential cracks. Moreover, the specimens were examined to determine whether they contained enamel, pulp horns, or other structural components.

**Figure 2:**
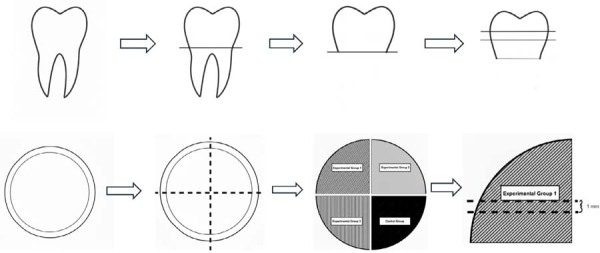
Schematic illustration of specimen preparation and allocation procedure

**Figure 3:**
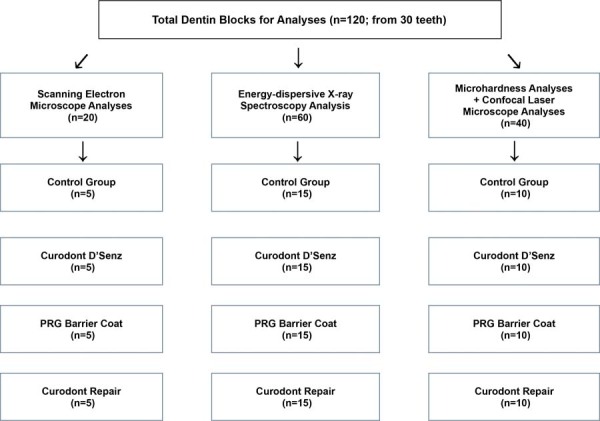
Schematic overview of specimen allocation and experimental design

Subsequently, the 120 dentin blocks were equally distributed into four groups (three experimental groups and one control group). From each tooth, one dentin specimen was randomly assigned to each group. Each dentin block was used exclusively for a single experimental group and no block contributed data to more than one group. The entire specimen preparation workflow, including root removal, crown sectioning, split-tooth design, and group allocation, is schematically illustrated in **Figures 2 and 3**.

### Application of test materials

To remove the smear layer and expose the dentinal tubules prior to material application, a 1% citric acid solution was prepared, and the dentin blocks were immersed in the solution for 30 seconds. Thereafter, the blocks were rinsed with distilled water and gently dried using sterile cotton pellets.^16^ The test materials were then applied to the surfaces of dentin specimens designated for microhardness, SEM, and EDS following the manufacturers' instructions (as detailed in **Figure 1**). For the CLSM analysis, 0.02 mg/g of 0.1% Rhodamine B dye was added to the test materials.^15^ The dye-labelled mixtures were then homogenized on a glass slab using a microbrush and applied to the dentin surfaces in accordance with the respective manufacturer guidelines. Following material application, each specimen was individually placed in a container filled with 10 mL of artificial saliva consisting of MgCl₂·6H₂O (0.148 mmol/L), K₂HPO₄ (4.59 mmol/L), KH₂PO₄ (2.38 mmol/L), KCl (8.39 mmol/L), calcium lactate (1.76 mmol/L), fluoride (0.05 ppm), sodium carboxymethylcellulose (2.25 mmol/L), and methyl-4-hydroxybenzoate (13.14 mmol/L), with the pH adjusted to 7.2 using KOH.^17^ and placed into an incubator (Nuve A.S., Izmir, Turkey) at 37°C and 1 atm pressure.

Dentin blocks that had been stored in artificial saliva at 37°C for 24 hours were then washed with distilled water, and 1-mm thick sections were obtained in the vertical direction using a precision cutting device. Dentin sections containing pulp horns and below 1-mm thick were excluded from this study. The obtained dentin sections were stored in sealed test tubes containing simulated body fluid in a light-proof environment until the application of the test methods.

### Assessment of Quantitative Analyses

In order to comparatively evaluate the effects of the tested materials on dentin tissue, four distinct analytical methods were employed. The surface microhardness of the dentin was assessed using the Vickers microhardness test, while changes in mineral content were analyzed via EDS. Surface morphological alterations and dentinal tubule occlusion were examined using SEM. Furthermore, the penetration depth of the materials and laser fluorescence values were measured by CLSM. For these assessments, each group comprised 10 specimens for microhardness testing and CLSM, five specimens for SEM analysis, and 15 specimens for EDS-based elemental analysis per material.

### Assessment of the Surface Microhardness

**Figure 4:**
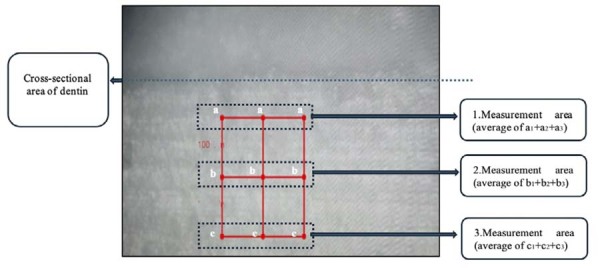
Schematic representation of the Vickers microhardness measurement protocol on the cross-sectional area of dentin

The surface microhardness values of the specimens were measured using a Vickers microhardness tester (Shimadzu Microhardness Tester HMV-2 Shimadzu Corporation; Kyoto, Japan) with a force of 30 g applied for 10 seconds. Hardness measurements were obtained from a total of nine different points on the surface of each specimen under 40x magnification: three points parallel to each other at 100-µm intervals and three points on the same plane. The mean of three measurements taken in a side-by-side configuration at 100-µm intervals 10 µm below the surface on which the material was applied was determined as the microhardness value of the surface at that depth (first measurement point) (**Figure 4**). In a similar manner, the mean microhardness values were computed for the second and third measurement points. Vickers hardness values were calculated using the standard Vickers formula based on the applied load and indentation diagonal lengths, and the results were initially expressed as N/mm^2^. All measurements were performed by the same researcher using the same calibrated device.

### SEM and EDS Analysis

**Figure 5:**
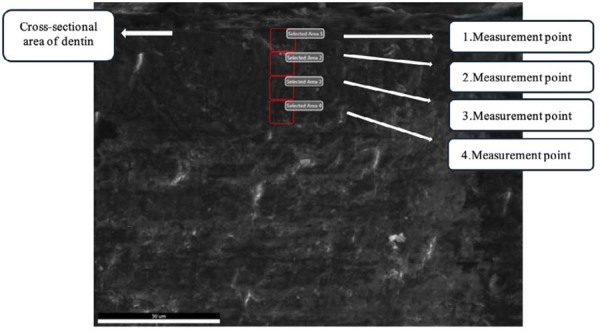
Representative cross-sectional SEM image illustrating the energy-dispersive X-ray spectroscopy (EDS) measurement protocol

The analysis was performed using a scanning electron microscope (Zeiss GeminiSEM 500, Carl Zeiss, Germany) equipped with an EDS detector (spot size: 5.0, accelerating voltage: 20.0 kV, analysis time: 60 s, ×2000 magnification) to assess mineral content and the dentin surface morphology. Prior to examination, possible elements were identified in the EDS system. Elemental composition was analyzed by measuring five 10×10-μm^2^ square areas in each specimen. Measurements were taken starting 10 µm below the dentin layer on which the material was applied, progressing in a straight line toward the pulp at 20-µm intervals from four areas (for a total of four measurement points) (% weight) (**Figure 5**). All parameters were standardized between study groups. Measurements were repeated thrice. Ca, P, and F content and the Ca/P ratio on the dentin surfaces were analyzed and the mean weight percentages of the elements were calculated.

In the process of scanning electron microscopy analysis, specimens were coated with a layer of gold measuring 30 nm in thickness and imaged at 10000x magnification. The Image J image processing and measurement tool was utilized to calculate the number and diameter of open tubules by means of the thresholding method (µm).

### CLSM Analysis

**Figure 6:**
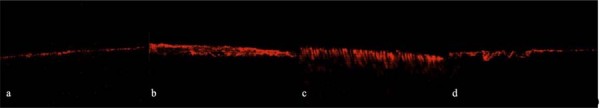
(a) Representative CLSM image (×20 magnification) showing dentin surface penetration in a specimen from the control group; (b) Representative CLSM image (×20 magnification) showing dentin surface penetration in a specimen from the Curodont™ D’Senz group; (c) Representative CLSM image (×20 magnification) showing dentin surface penetration in a specimen from the PRG Barrier Coat group; and (d) Representative CLSM image (×20 magnification) showing dentin surface penetration in a specimen from the Curodont™ Repair group.

Dentin sections were imaged using a helium-neon laser with a wavelength of 543 nm, scanned at x20 magnification starting approximately 10 µm below the surface on which the material was applied. The obtained images were transferred to a computer and analyzed using the Zeiss LSM Image Browser software (Version 4.2.0.121, Gottingen, Germany) on the Leica TCS-SPE system (Microsystems GmbH, Mannheim, Germany) (**Figure 6**). Dentine penetration depth was determined by measuring three areas in which maximum penetration was observed. These values were averaged. Changes in fluorescence intensity on the dentine surface were evaluated using ImageJ, an image analysis software (Wayne Rasband MD, National Institutes of Health, Bethesda, USA). In this analysis, a region of interest encompassing the entire dentin surface was defined on which the application was performed, and the laser fluorescence intensity within this area was quantitatively analyzed (pixels^2^).

### Statistical Analyses

Statistical analyses were conducted on SPSS 21.0 (IBM Corp., Armonk, NY, USA). Prior to statistical analyses, data distribution was assessed for normality using the Shapiro–Wilk test and for homogeneity of variances using Levene’s test. The parametric or non-parametric tests were chosen based on these assumptions. To compare the groups, the one-way analysis of variance (ANOVA) followed by the Tukey’s post hoc test was used for Vickers microhardness data, whereas the repeated measures ANOVA with the Bonferroni correction was applied for intra-group comparisons. The elemental data from the EDS analysis were evaluated by a one-way ANOVA procedure contingent upon the satisfaction of the criteria pertaining to normality and homogeneity of variances. In instances in which variances were found to be unequal, the Brown-Forsythe test was employed. Conversely, if the data failed to satisfy the assumptions of normality, the Kruskal-Wallis test was utilized. Subsequent post-hoc comparisons were conducted employing either Tukey’s or Bonferroni adjustments depending on the circumstances. To analyze intra-group comparisons involving repeated measures, the appropriate statistical test was employed, namely either repeated measures ANOVA or Friedman’s test, depending on the data distribution. For CLSM data (penetration depth and laser fluorescence), the Brown-Forsythe test was employed due to the violation of variance homogeneity, followed by Tamhane’s T2 post hoc test. For the analysis of SEM data, the Brown-Forsythe and Tamhane’s T2 tests were employed for variables that showed a normal distribution but were found to be non-homogeneous in terms of variance. In instances with non-normal variables, the Kruskal-Wallis and Bonferroni correction methods were implemented.

## Results

### Surface microhardness

The microhardness values obtained from the dentin specimens are shown in **Table 1**. In intergroup comparisons, the experimental groups showed significantly higher microhardness values than the control group (p<0.001), whereas no statistically significant difference was observed between the materials (p>0.05). The PRG Barrier Coat group had the highest mean microhardness value in the first measurement point (70.6±2.4), whereas the control group showed the lowest value in the third measurement region (54.2±3.3). Statistical analysis of the correlation between various measurement points for each material indicated statistically significant differences in the mean microhardness values across the first, second, and third measurements in all study groups (Control Group p=0.008; Curodont™ D'Senz p=0.001; PRG Barrier Coat p=0.001; Curodont™ Repair p=0.001), with a significant decrease in microhardness as measurement depth increased.

**Table 1:** Surface microhardness of specimens in different treatment groups (Newton/mm2) (mean ± SD)

	1. Measurement Point	2. Measurement Point	3. Measurement Point		Overall Mean of All Measurement Points
	Mean±St.Deviation	Mean±St.Deviation	Mean±St.Deviation		Mean±St.Deviation
**Control**	57.2±2.0^**a, A**^	55.9±2.5 ^**a, AB**^	54.2±3.3 ^**a, B**^	*p*=0.008	55.8±2.^**a**^
				F=6.404^§^	
**Curodont**^**TM**^ **D’Senz**	70.3±2.1^**b, A**^	64.7±2.5^**b, B**^	61.1±2.4^**b, C**^	*p*<0.001	65.4±2.2^**b**^
				F=236.163^§^	
**PRG Barrier Coat**	70.6±2.4^**b, A**^	65.5±2.1^**b, B**^	60.9±2.3 ^**b, C**^	*p*<0.001	65.7±1.8^**b**^
				F=81.649^§^	
**Curodont**^**TM**^ **Repair**	69.0±1.9^**b, A**^	64.5±2.2^**b, B**^	60.3±3.1^**b, C**^	*p*<0.001	64.6±2.0^**b**^
				F=61.370^§^	
***p***	*p*<0.001	*p*<0.001	*p*<0.001		*p*<0.001
	F=91.806	F=38.040	F=13.841		F=53,956

*Statistically significant differences between measurement points are indicated by uppercase letters (p < 0.05).

*Statistically significant differences between groups are indicated by lowercase letters (p < 0.05).

*F: ANOVA test statistic

*§: Repeated Measures ANOVA

### EDS

The Ca, P, and F contents and Ca/P ratios obtained from the dentin sections are shown in **Tables 2–6**. Statistically significant differences in Ca and P ion concentrations were observed between the groups (Ca: p=0.040; P: p=0.047). Overall, the PRG Barrier Coat group showed the highest mean Ca and P concentrations across all measurement points, and this difference is significant at first and second measurement points (p<0.05). In intragroup comparisons, statistically significant differences were detected among measurement points in the control (Ca: p<0.001; P: p<0.001) and Curodont™ D’Senz groups (Ca: p=0.003; P: p<0.001), with a notable increase in Ca and P ion levels as depth increased. In contrast, no significant differences were found between the measurement points in the PRG Barrier Coat group (Ca: p=0.293; P: p=0.087). Although statistically significant differences were observed overall in the Curodont™ Repair group (Ca: p=0.041; P: p=0.01), pairwise comparisons with Bonferroni correction showed no significant differences.

**Table 2:** Weight percentages of Ca content of specimens according to the treatment group obtained from Energy-Dispersive X-ray Spectroscopy analysis (mean ± SD)

	1. Measurement Point Mean±St.Deviation	2. Measurement Point Mean±St.Deviation	3. Measurement Point Mean±St.Deviation	4. Measurement Point Mean±St.Deviation	*p*
**Control**	20.1±5,9^**a, A**^	25.1±10.1^**ab, AB**^	26.0±10.0^**a, B**^	29.2±10.5^**a, B**^	*p*<0.001
					F=10.417^§^
**Curodont**^**TM**^ **D’Senz**	17.5±6.1^**a, A**^	21.0±7.2^**a, A**^	23.6±7.5 ^**a, B**^	24.0±7.7^**a, B**^	*p*=0.003
					F=9.542^§*^
**PRG Barrier Coat**	26.9±7.2^**b, A**^	28.2±5.8^**b, A**^	28.5±6.3^**a, A**^	29.0±6.3^**a, A**^	*p*=0.293
					F=1.283^§*^ GG
**Curodont**^**TM**^ **Repair**	19.0±9.4^**a, A**^	22.9±4.2^**ab, A**^	25.5±6.7^**a, A**^	24.9±6.6^**a, A**^	*p*=0.041
					F=3.799^§*^
***p***	*p*=0.004	*p*=0.047	*p*=0.380	*p*=0.171	
	H=13.619	F=2.819	F=1.045	F=1.733	

*Statistically significant differences between measurement points are indicated by uppercase letters (p < 0.05).

*Statistically significant differences between groups are indicated by lowercase letters (p < 0.05).

*F: ANOVA test statistic, H: Kruskal-Wallis, §: Repeated Measures ANOVA, §*:Greenhouse-Geiser

**Table 3:** Weight percentages of P content of specimens according to the treatment group obtained from Energy-Dispersive X-ray Spectroscopy analysis (mean ± SD)

	1. Measurement Point Mean±St.Deviation	2. Measurement Point Mean±St.Deviation	3. Measurement Point Mean±St.Deviation	4. Measurement Point Mean±St.Deviation	*p*
**Control**	8.90±3.03^**a, A**^	11.24±4.07^**a, B**^	11.91±3.66^**a, B**^	12.97±4.04^**a, B**^	*P<0.001*
					F=12.285^§*^
**Curodont**^**TM**^ **D’Senz**	8.11±2.75^**a, A**^	10.51±2.61^**a, B**^	11.12±2.77^**a, BC**^	11.55±2.7^**a, C**^	*p*<0.001
					F=22.364^§*^
**PRG Barrier Coat**	12.09±2.34^**b, A**^	12.84±1.58^**a, A**^	12.82±1.79^**a, A**^	13.09±1.65^**a, A**^	*p*=0.087
					F=2.655^§*^
**Curodont**^**TM**^ **Repair**	8.84±3.22^**a, A**^	10.31±3.10^**a, A**^	11.73±2.39^**a, A**^	11.44±2.30^**a, A**^	*p*=0.010
					F=5.568^§*^
***p***	*p=0.002*	*p= 0.095*	*p=0.407*	*p=0.229*^*¥*^	
	*F=5.783*	*F=2.227*	*F=0.985*		

*Statistically significant differences between measurement points are indicated by uppercase letters (p < 0.05).

*Statistically significant differences between groups are indicated by lowercase letters (p < 0.05).

*F: ANOVA test statistic, ¥: Brown-Forsythe §: Repeated Measures ANOVA, §*:Greenhouse-Geiser

**Table 4:** Weight percentages of F content of specimens according to the treatment group obtained from Energy-Dispersive X-ray Spectroscopy analysis (mean ± SD)

	1. Measurement Point	2. Measurement Point	3. Measurement Point	4. Measurement Point	*p*
	Mean±St.Deviation	Mean±St.Deviation	Mean±St.Deviation	Mean±St.Deviation	
**Control**	2.18±1.11^**a, A**^	2.14±1.64^**a, A**^	2.27±2.03^**a, A**^	1.81±1.64^**a, A**^	*p*=0.059 ^ѱ^
**Curodont**^**TM**^ **D’Senz**	2.51±0.79^**a, A**^	2.19±1.08^**a, A**^	2.13±0.92^**a, A**^	2.08±1.03^**a, A**^	*p*=0.127
					F=2.011^§^
**PRG Barrier Coat**	1.81±0.60^**a, A**^	1.96±0.64^**a, A**^	1.79±0.61^**a, A**^	1.98±0.53^**a, A**^	*p*=0.358
					F=1.105^§^
**Curodont**^**TM**^ **Repair**	1.89±0.64^**a, AB**^	1.96± 0.81^**a, A**^	1.19± 1.01^**a, B**^	1.64±0.78^**a, AB**^	*p*=0.013
					F=4.019^§^
***p***	*p*=0.089	*p*=0.547	*p*=0.111	*p*=0.285	
	F=2.278	H=2.122	F=2.181^¥^	H=3.790	

*Statistically significant differences between measurement points are indicated by uppercase letters (p < 0.05).

*Statistically significant differences between groups are indicated by lowercase letters (p < 0.05).

*F: ANOVA test statistic, H: Kruskal Wallis Test, ¥: Brown-Forsythe §: Repeated Measures ANOVA, ѱ:Friedman Test

**Table 5:** The Calcium/Phosphate (Ca/P) Ratio of Dentin according to the treatment groups (mean ± SD)

	1. Measurement Point Mean±St.Deviation	2. Measurement Point Mean±St.Deviation	3. Measurement Point Mean±St.Deviation	4. Measurement Point Mean±St.Deviation	*p*
**Control**	2.08±0.20^**a, A**^	2.10±0.37^**a, AB**^	2.13±0.30^**a, BC**^	2.24±0.37^**a, C**^	*p*=0.001^ѱ^
**Curodont**^**TM**^ **D’Senz**	2.23±0.91^**a, A**^	2.00±0.58^**a, A**^	2.12±0.47^**a, A**^	2.08±0.55^**a, A**^	*p*=0.233 ^ѱ^
**PRG Barrier Coat**	2.19±0.26^**a, A**^	2.17±0.25 ^**a, A**^	2.20±0.28^**a, A**^	2.18±0.26^**a, A**^	*p*=0.978 ^ѱ^
**Curodont**^**TM**^ **Repair**	2.14±0.53^**a, A**^	2.45±1.05^**a, A**^	2.21±0.67^**a, A**^	2.21±0.64^**a, A**^	*p*=0.457 ^ѱ^
***p***	*p*=0.305	*p*=0.129	*p*=0.788	*p*=0.809	
	H=3.622	H=5.672	H=1.057	F=0.322	

*Statistically significant differences between measurement points are indicated by uppercase letters (p < 0.05).

*Statistically significant differences between groups are indicated by lowercase letters (p < 0.05).

*F: ANOVA test statistic, H: Kruskal Wallis Test, ¥: Brown-Forsythe §: Repeated Measures ANOVA, ѱ:Friedman Test

**Table 6:** Weight percentages of Ca, P, F content, and Ca/P ratio of overall mean of all measurement points of the specimens according to the treatment group obtained from Energy-Dispersive X-ray Spectroscopy analysis (mean ± SD)

	Overall Mean of All Measurement Points							
	Ca	*p*	P	*p*	Ca/P	*p*	F	
	Mean		Mean		Mean		Mean	*p*
	±St.Deviation		±St.Deviation		±St.Deviation		±St.Deviation	
**Control**	25.1±8.4^**ab**^	*p*=0.040	11.25±3.33^**ab**^	*p*=0.047	2.14±0.26^**a**^	*p*=0.362	2.10±1.47^**a**^	*p* =0.098
**Curodont**^**TM**^ **D’Senz**	21.5±6.3^**a**^	F*=*2.949	10.32±2.49^**a**^	F=2.830	2.11±0.57^**a**^	H=3.196	2.22±0.84^**a**^	H=6.286
**PRG Barrier Coat**	28.2±5.8^**b**^		12.70±1.63^**b**^		2.19±0.24^**a**^		1.88±0.51^**a**^	
**Curodont**^**TM**^ **Repair**	23.1±4.8^**ab**^		10.57±2.06^**ab**^		2.25±0.43^**a**^		1.67±0.56^**a**^	

*Statistically significant differences between groups are indicated by lowercase letters (p < 0.05).

*F: ANOVA test statistic, H: Kruskal Wallis Test

Analysis of the Ca/P ratio showed no statistically significant differences between measurement points within the Curodont™ D’Senz (p=0.233), PRG Barrier Coat (p=0.978), and Curodont™ Repair (p=0.457) groups. Intergroup comparisons at each measurement point (1–4) and of overall group means also showed no significant differences (p=0.362). Similarly, fluoride ion concentrations neither differed significantly among groups across all measurement points (p=0.098) nor individual measurement points showed significant differences when compared to the control (p>0.05).

### SEM

The measurements of open dentin tubule diameter and count, average open tubule area, and total open dentin tubule area are shown in **Table 7**. A statistically significant difference was found in the comparison of all parameters between the groups (p<0.05). The PRG Barrier Coat group showed the lowest values for tubule number, diameter, average open tubule area, and total open dentin tubule area, whereas the control group showed the highest values across all parameters. Although the Curodont™ D’Senz and Curodont™ Repair groups showed lower values than the control group, the differences between these three groups were statistically insignificant (p>0.05).

**Table 7:** Mean and standard deviation values for the number and diameter of dentin tubules, open dentin tubules area, and total dentin tubules area (µm, µm2) (mean ± SD)

	Number of Dentin Tubules	Diameter of Dentin Tubules	Average Open Dentin Tubules Area	Total Open Dentin Tubules Area
	Mean	Mean	Mean	Mean
	±Standard Deviation	±Standard Deviation	±Standard Deviation	±Standard Deviation
**Control**	108.6±0.9^**a**^	3,02±0.51^**a**^	15.55±0.12^**a**^	1686.81±0.18^**a**^
**Curodont**^**TM**^ **D’Senz**	28.4±7.6^**ab**^	2.41±4.38^**ab**^	3.89±2.40^**ab**^	129.06±77.47^**b**^
**PRG Barrier Coat**	4.0±3.9^**b**^	0.77±0.57^**b**^	1.26±0.70^**b**^	7.85±8.32^**c**^
**Curodont**^**TM**^ **Repair**	35.2±12.2^**ab**^	2.24±0.25^**ab**^	2.23±0.68^**ab**^	78.77±24.01^**b**^
***p***	*p*=0.001	*p*=0.007	*p*=0.002	*p*<0.001*
	H=16.260	H=12.257	H=15.137	

*Statistically significant differences between groups are indicated by lowercase letters (p < 0.05).

*H: Kruskal Wallis Test, *Forsythe Analyses

**Figure 7:**
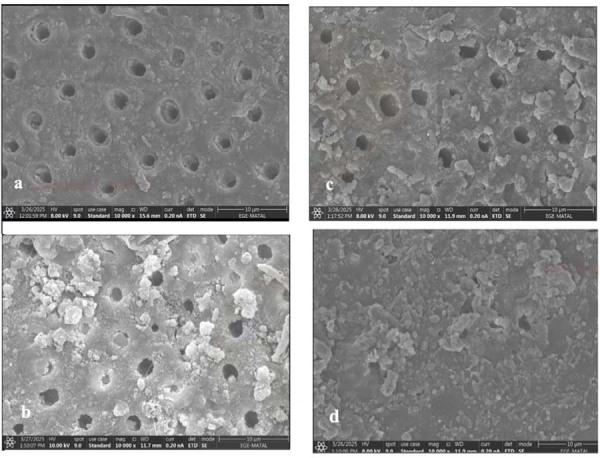
Representative scanning electron microscopy (SEM) images of dentin surfaces obtained at ×10,000 magnification after treatment with several agents. (a) Control group: dentin surface showing completely open dentinal tubules with no evidence of tubule occlusion; (b) Curodont™ D’Senz group: presence of an amorphous surface layer with partial occlusion of some dentinal tubules; (c) Curodont™ Repair group: dentin surface showing small crystal-like deposits around predominantly open dentinal tubules; (d) PRG Barrier Coat group: dentin surface covered by a continuous and homogeneous superficial layer, with most dentinal tubules appearing uniformly and completely occluded.

SEM images obtained at ×10000 magnification are shown in **Figure 7**, illustrating the morphological changes induced by each treatment agent on the dentin surface. In the control group, dentinal tubules seemed completely open and highly permeable. In the Curodont™ D’Senz group, an amorphous surface layer and partial occlusion in some tubules were observed. In the Curodont™ Repair group, small crystal-like deposits were detected around open tubules. SEM images of dentin treated with PRG Barrier Coat showed a continuous superficial layer across the dentin surface, with most tubules appearing uniformly and completely occluded.

### CLSM

Data on penetration depth into the dentin surface and laser fluorescence values obtained by confocal laser scanning microscopy are shown in **Table 8**. The mean penetration depth values and laser fluorescence values in all experimental groups were found to be significantly higher than those in the control group (p<0.001). The PRG Barrier Coat and Curodont™ D’Senz groups showed the deepest penetration into the dentine, whereas the highest laser fluorescence intensity was detected in the PRG Barrier Coat group (p<0.05). The Curodont™ Repair group showed moderate values for both parameters, whereas the control group, the lowest values.

**Table 8:** The mean and standard deviation (±SD) values for dentin penetration depth and laser fluorescence (µm)

	Penetration Depth	Laser Fluorescence
	Mean	Mean
	±St.Deviation	±St.Deviation
**Control**	7.0±1.1^**a**^	6.1±1.2^**a**^
**Curodont**^**TM**^ **D’Senz**	37.6±3.2^**b**^	30.8±7.2^**bc**^
**PRG Barrier Coat**	36.5±3.5^**b**^	45.4±15.6^**b**^
**Curodont**^**TM**^ **Repair**	16.9±1.3^**c**^	26.0±7.4^**c**^
***p***	*p*<0.001	*p*<0.001

*Statistically significant differences between groups are indicated by lowercase letters (p < 0.05).

*Brown-Forsythe Test

## Discussion

In this study, an *in vitro* model was employed to comparatively evaluate the effects of various therapeutic agents used for dentin hypersensitivity on dentin tissue. The materials were assessed regarding surface microhardness, elemental composition, dentin tubule occlusion, and penetration ability. According to the results of this study, the null hypothesis—stating that there would be no statistically significant differences between the effects of the tested agents on dentin tissue—was rejected as significant differences were observed between the effects of the tested materials.

In this study, the Vickers microhardness test was employed to assess changes in the hardness of dentin tissue. Measurements were taken from nine points, starting below the surface on which the material was applied and extending toward the pulp. Overall, three points were measured at 100 µm-intervals along the vertical axis, and three additional points were measured on a horizontal plane perpendicular to each of these vertical points. A load of 30 grams was applied for 10 seconds. This approach aimed to evaluate the hardness profile regarding depth and the horizontal plane.

The microhardness analyses in our study showed that the microhardness values of the control group were significantly lower than those of all other material groups, whereas no statistically significant difference was observed between the Curodont™ D’Senz, PRG Barrier Coat, and Curodont™ Repair groups. The fact that higher microhardness values were obtained in all measurement areas of the surfaces in which the materials were applied suggests that these agents strengthen the dentin surface or increase its mineral content. This finding is consistent with other studies reporting that bioactive glasses and peptide-based remineralization agents promote apatite formation on the dentin surface and may increase microhardness.^17-20^When the changes created by the materials on the surfaces on which they were applied were separately evaluated, it was found that microhardness values statistically and significantly decreased from the first measurement region to the third measurement one (from the surface to the depth) in all groups. The similar hardness profile observed in all materials (first measurement region > second measurement region > third measurement region) is consistent with the regional effects of remineralizing agents in the literature.

Although no statistically significant differences were found between the average microhardness values of all measurement points after the application of the tested materials, the PRG Barrier Coat group showed a slight superiority over the other materials. Its notably higher microhardness values observed in its first measurement point (closest to the surface) can be attributed to the rapid mineralization capacity of bioactive glass particles.^22^In the literature, the silicate, fluoride, and strontium ions contained in PRG Barrier Coat have been reported to support remineralization of dental tissues and enhance acid resistance.^23^ Sauro et al. also reported that resin systems containing bioactive glass promote apatite formation at the dentin interface and significantly improve hardness and elasticity.^24^Furthermore, a study that combined microhardness, SEM, and EDS analyses showed that this material achieves effective remineralization by buffering the acidic environment via ion release.^25^These ionic interactions may contribute to mineral accumulation on the dentin surface, thereby potentially enhancing its mechanical properties—a finding consistent with the increased microhardness values in our study.

In this study, dentin specimens treated with the peptide-based biomimetic agents Curodont™ D’Senz and Curodont™ Repair showed higher microhardness values than the control group. Although the effect of Curodont™ D’Senz on dentin hardness has not been directly studied in the literature, findings from research on P₁₁-4 peptides suggest that these molecules support mineral formation, particularly on the outer dentin layers. Studies by Alkilzy using CLSM and SEM showed that mineral deposition driven by P₁₁-4 tends to be concentrated near the surface, decreasing with depth.^19^ Supporting that observation, Danisman et al. reported the formation of apatite-like minerals on enamel surfaces following the use of Curodont™ Repair, with mineral density gradually decreasing in deeper areas.^26^ These findings are in line with our microhardness results, which also showed a depth-dependent decrease in hardness.

Ca and P are the basic components of the hydroxyapatite crystal structure and the main indicators of the degree of mineralization in hard tooth tissues such as dentine and enamel. Therefore, in this study, Ca, P, Ca/P ratio, and F content were analyzed using EDS to evaluate the mineral profile of dentin tissue. The findings showed statistically significant differences in Ca and P ion concentrations between groups in the first and second measurement points from the surface toward the pulp. In the first and second measurement regions, significantly higher Ca levels were observed in the PRG Barrier Coat group than in the other groups; whereas similar Ca levels were recorded in the control, Curodont™ D’Senz, and Curodont™ Repair groups. Although the differences between groups decreased in the third and fourth measurement points, the PRG Barrier Coat group consistently showed the highest ion concentrations and a constant and homogeneous ion profile at all measurement depths.

The findings of this study suggest that PRG Barrier Coat may contribute to the mineralization of the dentin surface due to its bioactive glass content. Its high levels of Ca and P are thought to result from the high ion release capacity of the material, which is attributed to its S-PRG content. Studies involving fissure sealants containing S-PRG have reported that these agents release ions at significantly higher levels than other materials, particularly within the first 24 hours.^22^ Moreover, it has been shown that S-PRG-containing agents penetrate dentin tissue by releasing ions from their structure after application to the dentin surface.^27^ Remineralization studies on demineralized primary teeth have reported that PRG Barrier Coat supports mineralization by increasing their Ca and P content.^34,35^ Similarly, another study found that PRG Barrier Coat prevents dentin demineralization, inhibits biofilm formation, and promotes remineralization.^8^ These findings in the literature support the homogeneous ionic increase profile in our study based on EDS data, as well as the high levels of Ca, P, and F, and the Ca/P ratios after 24 hours. Furthermore, the microhardness analysis results are consistent with these ionic changes and confirm the remineralization capacity of PRG Barrier Coat.

In this study, higher levels of Ca, P, and F were observed in the PRG Barrier Coat group, whereas lower mineral values were detected in the Curodont™ Repair and Curodont™ D'Senz groups. According to the literature, Curodont™ D’Senz is reported to prevent mineral loss and support mineral gain thanks to its content of Ca, P, F, and P₁₁-4 peptide.^30^ In a study evaluating the effects of Curodont™ Repair and Curodont™ D'Senz on dentin, it was stated that these materials achieved Ca and P peak increases comparable to those of NaF and CPP-ACP.^37^In this study, the effectiveness of the materials was analyzed after 24-hour incubation in artificial saliva to evaluate their short-term effects in relieving dentin hypersensitivity-related symptoms. The observed discrepancy between our results and those of previous studies may be attributed to the fact that the biomimetic effect of self-assembling peptides requires a longer period to manifest. In a study in which 37% orthophosphoric acid was applied to the dentin surface prior to Curodont™ Repair, it was suggested that the self-assembly mechanism of P11-4 is pH-sensitive and that the low-pH environment created by acid etching supports peptide fiber assembly and the formation of a stable scaffold.^10^ This mechanism may contribute to the strengthening of dentin via interactions with Ca and P ions. It has been suggested that P₁₁-4 fibers may stabilize collagen fibrils by hydroxyapatite formation, potentially protecting against proteolytic degradation.^10^ However, the complete formation of fibril networks necessitates approximately 24 hours, and the implementation of repeated applications over a period of 3–6 months may be required for effective remineralization.^32^ In this context, the limited effects of the peptide-based materials in this study may be attributed to the incomplete formation of the self-assembled fibrous network within 24 hours. Since the self-assembly process and subsequent mineral deposition are time-dependent, the short evaluation period may have failed to enable the sufficient maturation of the peptide scaffold to achieve optimal remineralization and tubule occlusion. This may explain the relatively lower mineral-related values, less homogeneous surface morphology, and reduced tubule occlusion in the peptide-based groups when compared to PRG Barrier Coat. Moreover, in this study, to mimic dentin hypersensitivity and expose the dentinal tubules by removing the smear layer, all specimens were treated with 1% citric acid. Moreover, according to the manufacturer’s instructions, 37% orthophosphoric acid was applied for Curodont™ Repair and 6% citric acid for Curodont™ D’Senz as part of surface preparation. Due to the demineralization potential of these two acids, the removal of Ca and P ions from the surface may have contributed to the lower elemental content in these groups when compared to the PRG Barrier Coat group. Moreover, it should be noted that a standardized 1% citric acid pre-treatment was applied to all specimens before the manufacturer-specific conditioning procedures to ensure uniform smear layer removal and comparable baseline dentin conditions across all groups. This approach was intended to improve standardization of the experimental model. However, the combined effect of the initial citric acid application and the subsequent material-specific surface treatments may also have influenced dentin surface characteristics and, consequently, material performance.

In this study, in addition to the elemental content of the materials, the morphological changes they caused on the dentin surface and tubule structure were also analyzed using SEM. The surface morphology analyses on the obtained images showed that the applied materials caused significant structural changes on the dentin surface when compared to the control group. In the group treated with PRG Barrier Coat, surfaces were covered with a homogeneous and continuous layer, whereas, in the groups treated with Curodont™ D’Senz and Curodont™ Repair, irregular surface structures were observed in some areas, and mineral accumulation was distributed more heterogeneously. In contrast, no protective layer formation was observed on the surface of the control group, and the structure showed a weak appearance regarding mineralization. The literature contains conflicting findings regarding the ability of Curodont™ D’Senz to occlude dentinal tubules. For example, Zuluaga Morales^31^ (2023) reported that, based on SEM analysis, some tubules were occluded but mineralization was irregular, whereas Mosleh and Eltayeb^33^ (2022) reported that the material was insufficient for tubule occlusion and unable to form a homogeneous surface layer. These results suggest that the material may contribute to dentinal tubule occlusion and structural integrity.^10^ The irregular and heterogeneous mineral deposition in our study suggests that the efficacy of Curodont™ D’Senz may depend on variables such as application time or surface preparation. Similarly, irregular mineral deposition on the dentin surface and the absence of a homogeneous layer were observed in the Curodont™ Repair group. Previous studies supported by SEM analyses reported that this material supports remineralization but shows partial tubule occlusion and heterogeneous mineral distribution.^31,35^ These findings suggest that, despite their biomimetic potential, Curodont™ Repair and Curodont™ D’Senz are ineffective at forming a stable, homogeneous protective layer in the short term *in vitro*, which is consistent with the surface irregularities in our study. In our study, unlike the peptide-based materials, a homogeneous layer covering the dentin surface was observed in the group treated with PRG Barrier Coat, indicating that the material can form an effective physical barrier. This finding is consistent with other studies reporting that PRG Barrier Coat homogeneously occludes dentinal tubules, supports mineralization, and reduces surface permeability.^11,18^ Furthermore, our microhardness and EDS analysis results also support the SEM findings, showing that this material provides a strong barrier and promotes remineralization in the treatment of dentin hypersensitivity.

Dentin tubules are microscopic canal systems that extend throughout the dentin matrix. Their density, diameter, and cross-sectional area may vary depending on anatomical location, pathological conditions (e.g. erosion and wear), and the properties of the applied chemical or biofunctional materials. This study evaluated the effects of applied agents on dentin microstructure by analyzing the number, diameter, and area of dentin tubules. The data showed that all treatment groups produced significant structural changes in micromorphology when compared to the control group. The control group showed the highest number, diameter, and open tubule area, whereas the PRG Barrier Coat group showed statistically significantly lower values for all these parameters. In the Curodont™ D'Senz and Curodont™ Repair groups, the number, diameter, and area of open tubules were significantly reduced when compared to the control group. However, no difference was observed between these two groups. Following the application of the PRG Barrier Coat, a homogeneous and continuous layer was observed to cover the dentin surface, and the diameter of the tubules and the open tubule area were found to be significantly reduced. These results suggest that the material may contribute to dentinal tubule occlusion, providing structural integrity thanks to its S-PRG content. The material can also create a physical barrier for controlling dentinal sensitivity. Ribeiro, et al.^35^ (2025) confirmed that using SEM that PRG Barrier Coat provides compact and regular tubule occlusion. Similarly, another study has reported that this material significantly reduces dentin permeability and forms a compact protective layer.^37^ In a study under simulated oral environmental conditions, it was showed that PRG Barrier Coat maintains its tubule occlusion efficacy even in the presence of environmental stress factors.^5^ Mosquim, et al.^37^ (2022) also reported that PRG Barrier Coat forms a thicker and more complete layer than other agents after acid exposure. These findings are consistent with our study, which showed that PRG Barrier Coat provides tubule occlusion on the dentin surface by forming a homogeneous protective layer. The light-curable, resin-based structure of the material enables stronger bonding to the surface and long-term efficacy than non-curable, biofunctional agents, such as Curodont™ D'Senz and Curodont™ Repair. However, the limited tubule occlusion in the Curodont™ Repair and Curodont™ D'Senz groups is thought to relate only to the 24-hour effect of the materials.^35^ Zuluaga Morales^31^ (2023) reported partial occlusion and heterogeneous mineral distribution in tubules following the application of Curodont™ D’Senz. Mosleh and Eltayeb^33^ (2022) showed similar limited efficacy of this material in reducing dentinal permeability. These findings are consistent with the SEM observations in our study. Similar conflicting findings exist for Curodont™ Repair. Zuluaga Morlaes^31^ (2023) reported that most tubules remained open and that mineralization was distributed irregularly, whereas Barbosa-Martins, et al.^34^ (2018) observed differences in tubule opening density. These discrepancies may be due to the variability of the biomimetic effects of peptide-based agents, which depend on environmental conditions and application duration and frequency. The literature reports that self-assembling peptides form three-dimensional fibril-like structures that support hydroxyapatite crystallization and promote mineral deposition in subsurface regions over time.^30,38^ For example, limited tubule narrowing was observed with a single dose of sADP5 peptide, whereas regular mineral accumulation and complete tubule obstruction were reported following three repeated applications.^38^ In this context, the limited tubule occlusion observed after a single application in the Curodont™ D'Senz and Curodont™ Repair groups in our study can be attributed to the cumulative effects of these agents and the time-dependent nature of the mineralization process. Compared to PRG Barrier Coat, the remineralization process starts later but may be effective in the long term.

In our study, the penetration depth and density of the materials into dentinal tubules were evaluated using CLSM. As a result of this analysis, statistically significant greater penetration depths were observed in the PRG Barrier Coat and Curodont™ D’Senz groups when compared to the control group, whereas the Curodont™ Repair group showed a moderate level of penetration. The observed performance of PRG Barrier Coat may be related to its material composition, including the presence of triethylene glycol dimethacrylate. Its low molecular weight may facilitate deep penetration into dentinal tubules and homogeneous polymerization, increasing the effectiveness of the material. Similar to PRG Barrier Coat, a high penetration depth was also observed in the Curodont™ D’Senz group. According to the manufacturer, the product occludes dentinal tubules within two minutes and provides an effective barrier for up to three months. However, no specific study in the literature has directly evaluated the penetration capacity of this material. Nevertheless, surface-level SEM studies have shown that Curodont™ D’Senz has at least a partial effect in reducing dentin permeability. Although these studies provide no information on penetration depth, the high penetration depth in this study may indicate a potential for improved tubule interaction. Another parameter in this study using CLSM refers to the fluorescence signals reflecting the penetration density of the materials into the dentinal tubules. The results showed that higher fluorescence values were observed in the Curodont™ Repair and Curodont™ D’Senz groups when compared to the control group. However, the highest value was recorded in the PRG Barrier Coat group. Since fluorescence intensity reflects the density of material penetration within dentinal tubules, the higher values in the PRG Barrier Coat group indicate a more homogeneous and dense distribution of the material within the tubule structure. This finding is consistent with the SEM results that show more effective tubule occlusion, as well as with the mineral changes and penetration depth data, suggesting that deeper and denser penetration may contribute to the superior sealing ability of this material. The laser fluorescence values from the self-assembling peptide-based systems support the view that such systems have a limited effect in relieving dentin hypersensitivity in the short term.

Overall, the findings of this study demonstrate that the tested materials showed different mechanisms and levels of effectiveness in modifying dentin structure within its short-term evaluation period. While all materials influenced tubule occlusion, mineral-related parameters, and penetration behavior to varying extents, differences in their mode of action seem to play a critical role in their observed performance. In particular, the relatively limited effects in the peptide-based groups may be associated with the time-dependent nature of their mechanism of action. Considering these findings, the 24-hour experimental design in this study may have underestimated the full potential of these materials. Therefore, longer observation periods and repeated application protocols should be considered in future studies to better evaluate their cumulative effects on dentin remineralization and dentinal tubule occlusion. Moreover, *in vivo* studies are needed to determine whether these effects translate into clinically relevant outcomes.

## Conclusion

Within the limitations of this *in vitro* study, PRG Barrier Coat showed higher short-term efficacy than peptide-based biomimetic agents regarding dentinal tubule occlusion, mineral-related parameters, and penetration behavior. While Curodont™ D’Senz and Curodont™ Repair showed promising effects, their performance was less pronounced within 24 hours. From a clinical perspective, materials providing rapid tubule occlusion and effective surface sealing may be preferred for immediate symptom relief, whereas peptide-based biomimetic agents may be more suitable for long-term management due to their time-dependent remineralization potential. Further long-term and clinical studies are required to confirm these findings.
